# Seeing the Forest through the (Phylogenetic) Trees: Functional Characterisation of Grapevine Terpene Synthase (*VviTPS*) Paralogues and Orthologues

**DOI:** 10.3390/plants10081520

**Published:** 2021-07-26

**Authors:** Samuel J. Smit, Melané A. Vivier, Philip R. Young

**Affiliations:** Department of Viticulture and Oenology, Faculty of AgriSciences, South African Grape and Wine Research Institute, Stellenbosch University, Stellenbosch 7600, South Africa; cobus.smit@york.ac.uk (S.J.S.); mav@sun.ac.za (M.A.V.)

**Keywords:** grapevine, terpene synthase, sesquiterpene, genotypic variation, gene structure

## Abstract

Gene families involved in specialised metabolism play a key role in a myriad of ecophysiological and biochemical functions. The *Vitis vinifera* sesquiterpene synthases represent the largest subfamily of grapevine terpene synthase (*VviTPS*) genes and are important volatile metabolites for wine flavour and aroma, as well as ecophysiological interactions. The functional characterisation of *VviTPS* genes is complicated by a reliance on a single reference genome that greatly underrepresents this large gene family, exacerbated by extensive duplications and paralogy. The recent release of multiple phased diploid grapevine genomes, as well as extensive whole-genome resequencing efforts, provide a wealth of new sequence information that can be utilised to overcome the limitations of the reference genome. A large cluster of sesquiterpene synthases, localised to chromosome 18, was explored by means of comparative sequence analyses using the publicly available grapevine reference genome, three PacBio phased diploid genomes and whole-genome resequencing data from multiple genotypes. Two genes, *VviTPS04* and -*10*, were identified as putative paralogues and/or allelic variants. Subsequent gene isolation from multiple grapevine genotypes and characterisation by means of a heterologous in planta expression and volatile analysis resulted in the identification of genotype-specific structural variations and polymorphisms that impact the gene function. These results present novel insight into how grapevine domestication likely shaped the *VviTPS* landscape to result in genotype-specific functions.

## 1. Introduction

The *Vitis vinifera* reference genome has been an invaluable resource for the identification of genes involved in metabolic processes of agronomic interest [1]. The genetic basis for various traits associated with flavour and aroma have increasingly been elucidated since the genome became available, as recently reviewed by reference [2]. A major contributor to flavour and aroma profiles are the terpenes, a chemically diverse class of metabolites, mainly associated with floral and Muscat aromas (for example, linalool, geraniol, nerol, α-terpineol, and hotrienol) or a pepper aroma (for example, rotundone) inferred by mono -and sesquiterpenes, respectively [2,3,4,5,6]. The near-homozygous reference genome PN40024 contains 152 *V. vinifera* terpene synthase (*VviTPS*)-like loci, of which 69 are predicted to be functional [1,7]. Thirty of these gene models were subsequently linked to a functional enzyme but were isolated from various genotypes [7].

The *VviTPS* family annotated on the PN40024 reference genome largely encodes for genes involved in mono- (C10) and sesquiterpene (C15) biosynthesis. These metabolites are biosynthesised via two compartmentalised pathways. The cytosolic mevalonate (MVA) and plastidial 2-C-methyl-D-erythritol 4-phosphate (MEP) pathways both result in the C5 terpene precursors isopentenyl diphosphate (IPP) and dimethylallyl diphosphate (DMAPP). These isomeric precursors are coupled in a head-to-tail manner by prenyl transferases that result in the mono-TPS substrate geranyl diphosphate (GPP) and sesqui-TPS substrate farnesyl diphosphate (FPP). FPP can be isomerised in a rate-limiting reaction to form the nerolidol diphosphate (NPP) substrate [8,9,10,11]. The array of terpenes produced by a single TPS varies greatly, from a single product to an excess of 40 terpenes [12,13,14,15]. This is due to how the TPS active site interacts with its substrate. Depending on how long the TPS can shield the reaction from being quenched, one will typically see the biosynthesis of acyclic and cyclic terpene structures, with the former usually seen due to premature/early quenching [11,16,17,18]. The added double bond in the C15 FPP/NPP substrates, relative to GPP, allows for more conformational changes to take place, catalysed by a sesqui-TPS, resulting in a more diverse array of chemical structures [14,19,20].

The near-homozygosity (~93%) of the PN40024 reference genome [1,21] does not reflect the structural complexity associated with commercial heterozygous diploid cultivars [22,23]. To address this limitation, recent sequencing efforts employed long-read genome sequencing that allows haplotype-aware genome assemblies [24,25,26]. Comparative analyses of the genomic landscape for the diploid genomes of Cabernet Sauvignon (CS), Chardonnay (CH), or Carménère (CR) [24,25,26] highlighted extensive genotype-specific structural variations (SV) within the *VviTPS* family [27]. The extensive duplication of *VviTPS* genes furthermore resulted in numerous genes having similar functions with various mechanisms potentially involved in these duplication events [7,20,28,29]. The physical proximity of related *VviTPS* subfamily members on a chromosome are indicative of tandem duplication events, whereas remnants of transposable elements suggest that transposon-mediated duplication allowed for the genome-wide movement of *VviTPS* genes [7]. Segmental duplications that are highly homologous (94% and >10 kb in size) contribute to 17% of the PN40024 genome, resulting in large repetitive genomic regions [30]. Domestication-driven introgression between germplasms has furthermore resulted in highly complex genomes that show greater heterozygosity in the domesticated varieties than the wild parent(s) [31,32,33,34].

Tandem *TPS* duplications often form multi-gene clusters of varying sizes but show similar catalytic mechanisms. In tomatoes (*Solanum lycopersicum*), for example, a sesqui-TPS cluster of six genes consists of two pseudogenes and characterised genes with divergent catalytic active sites [35]. In rice (*Oryza* spp.), the evolution of *TPS* gene clusters involved in insect defence shows species-specific cluster differences that go through a combination of duplication and subsequent functionalisation events. These gene clusters, furthermore, show species-specific expressions and emission patterns in response to insect infestations [36].

A 690-kb region of PN40024 chr18 contains 44/152 *VviTPS*-like loci, 20 encoding for putative sesquiterpene synthases (*VviTPS01-19* and *-30*). Eleven of these genes have been functionally characterised [7,20]. The annotation of the *VviTPS* families for CH, CS, and CR, however, revealed that there are potential structural differences in the genome space for the chr.18 cluster [27]. To date, the relatedness of the *VviTPS* paralogues in this cluster has not been explored.

Of particular interest is *VviTPS10*, a gene model linked to two distinct enzyme functions with the VvGwaBer nucleotide sequence being concordant to the gene model [7]. A genotype-specific homologue, VviMATPS10, however, showed various nonsynonymous mutations that resulted in a novel function for the cultivar Muscat of Alexandria [20]. The extent of these sequence differences presented two possibilities: *VviTPS10* has two alleles with unique functions or a highly conserved paralogue inferring the second function. *VviTPS04* was previously identified as a putative homologue or allelic variant of *VviTPS10* using the PN40024 reference genome [1,7,20].

In this study, a cluster of chromosome (chr.) 18 genes, which includes *VviTPS10* and *-04*, were analysed in order to explore the possible allelic and/or duplication differences. The annotated *VviTPS* landscape of three phased diploid genomes [27] was utilised in combination with the publicly available whole-genome resequencing (WGRS) data [37] to identify the structural differences in the selected genotypes. The candidate gene regions homologous to *VviTPS10* were subsequently isolated from multiple *V. vinifera* genotypes and functionally characterised by means of *Agrobacterium*-meditated transient gene expression in *Nicotiana benthamiana*, followed by a volatile terpene analysis using headspace solid-phase microextraction (HS-SPME) and gas chromatography-mass spectrometry (GC-MS). The active sites of isolated paralogues were also compared to identify amino acid residues that could result in possible genotype-specific enzyme functions.

## 2. Results

### 2.1. Isolated VviTPS04 and -10-Like Paralogues

**Figure 1:**
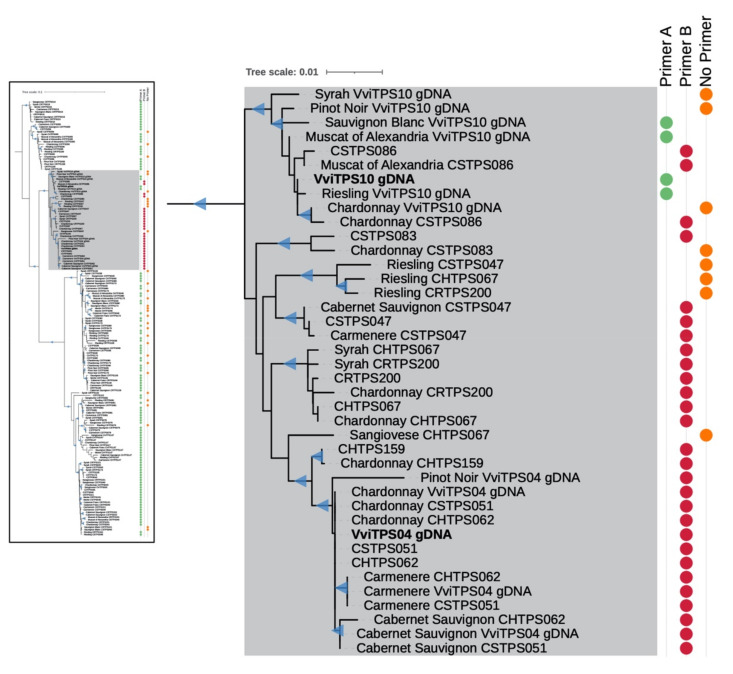
Phylogenetic tree of the consensus sequences assembled from the WGRS data [37] using *VviTPS* gDNA models predicted from phased diploid genomes [27] for the cultivars Chardonnay (CH) [24], Carménère (CR) [25], and Cabernet Sauvignon (CS) [26] and the grapevine reference genome PN40024 [1,7]. The PN40024 *VviTPS04* and *-10* gDNA models are indicated in bold. WGRS genotypes for which the gene models were predicted are shown, followed by the gDNA model [27] that the reads mapped to. The coloured circles show which primers bind at the 5′ terminal ends. Triangular symbols on the tree indicate high confidence branch nodes with an approximate likelihood-ratio test (SH-aLRT) >85% and ultrafast bootstrap support >95% using 1000 replicates.

The PN40024 genome showed that the forward strand of the *VviTPS04* locus had a start codon 27 bp upstream of the start codon for the *VviTPS10* locus [1,7]. These two loci had high homology with a total of 28 *VviTPS*-like loci from the three phased diploid genomes (8 in CH, 9 in CR, and 11 in CS), predicted by Smit et al. (2020). The 28 phased diploid and two PN40024 gDNA models (hereafter referred to as reference sequences) were subsequently used to map short reads from 11 different genotypes that were used in the WGRS effort by reference [37]. This resulted in 112/330 consensus sequences with near-complete coverage, with a further 35 that lacked coverage at the 5′ terminal region, relative to the reference sequence. Only a single reference sequence (*CRTPS105*) did not result in the recovery of at least a single consensus sequence from the 11 WGRS genotypes selected for this analysis. This resulted in 147 consensus and 30 reference sequences that were subsequently aligned and their phylogenetic relationships assessed, as shown in Figure 1 (a higher-resolution version of the complete tree is available in Supplementary Figure S1). The lowest pairwise identity in this alignment was 89.2%, with the highest being 99.5%, indicating that all sequences in the multiple sequence alignment (MSA) were highly similar. The phylogenetic tree revealed that *VviTPS04* and *VviTPS10*-like sequences were grouped into a distinct clade, coloured in grey in Figure 1. Eight of the sequences in this grey clade lacked coverage at the terminal end. The *VviTPS04*-like sequences (containing the additional 27 bp at the 5′ terminal end) could be separated further from the *VviTPS10*-like sequences, forming distinct subclades. The *VviTPS10* gDNA-containing subclade had an orthologous gDNA model from CS (*CSTPS086*) with eight gDNA models predicted from the WGRS data. The subclade containing *VviTPS04*-like sequences had two near-identical reference sequences (*CSTPS051* and *CHTPS062*) to *VviTPS04*. Ten gene models in this clade were predicted from the WGRS mapping. Incongruence between the PN40024 *VviTPS04* gene model and the gene model predicted for the Pinot noir TA-379 WGRS genotype was observed. The short branch lengths indicated high homology for sequences within the grey clade, despite the bifurcation for the *VviTPS04*-like and *VviTPS10*-like subclades. The remaining sequences were grouped into five distinct clades (Supplementary Figure S1). These sequences were near-identical at the 5′-region, with 24 lacking coverage. All 175 sequences in the MSA had a near-identical 3′-region. The high homology in the terminal ends meant that three primers could be designed to target 112/175 sequences: Primer A and B targeted the 27-bp difference at the 5′ region, while a single conserved 3′ primer was designed. These results are shown in Table 1, where groups A and B correspond to the binding of their respective forward primers, whereas group C reflects the sequences that lack coverage at these primer-binding regions. The primer-binding circles in the phylogenetic tree (Figure 1) use the same bin colours used in Table 1. Nucleotide sequences for the reference genes and those predicted by mapping of the WGRS data is available is Supplementary File S1.

**Figure 2:**
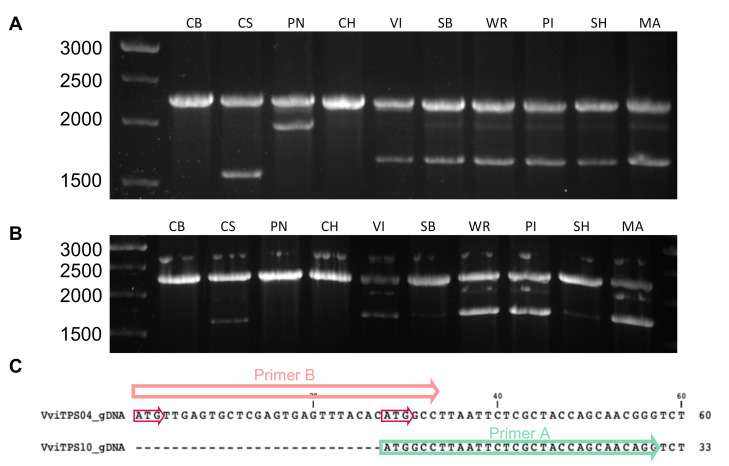
The gDNA PCR of nine *V. vinifera* genotypes using the conserved reverse primer in combination with primer A (**A**) or primer B (**B**). The following abbreviations were used for the respective genotypes: Cabernet Sauvignon (CS), Chardonnay (CH), Chenin Blanc (CB), Muscat of Alexandria (MA), Pinot noir (PN), Sauvignon Blanc (SB), Shiraz (SH), and Viognier (VI). The 27-bp difference in the *VviTPS04* and *VviTPS10* reference genome gene models with the binding positions of their respective forward primers are shown (**C**). The two in-frame start codons are indicated on *VviTPS04* by the red arrows.

It should be noted that the 27-bp difference results in two start codons being in frame, shown in Figure 2C. The primers were validated through PCR amplification using gDNA from nine genotypes, as shown in Figure 2A,B. The expected gDNA band of ±2260 bp was present in all genotypes, with certain genotypes showing a second band at ±1700 bp for primer set B (Figure 2B). A second band was notably absent for CH using either primer combination. For primer set A, a 2000-bp amplicon was observed in PN, with this band also faintly visible in six other genotypes (Figure 2A). Primer set A resulted in a unique second amplicon for CS. These results support the in silico predictions and highlighted the extensive homology between the VviTPS04 and VviTPS10-like loci.

### 2.2. Isolation and Sequence Comparison of VviTPS04 and -10 Homologs from Nine Grapevine Genotypes

**Figure 3:**
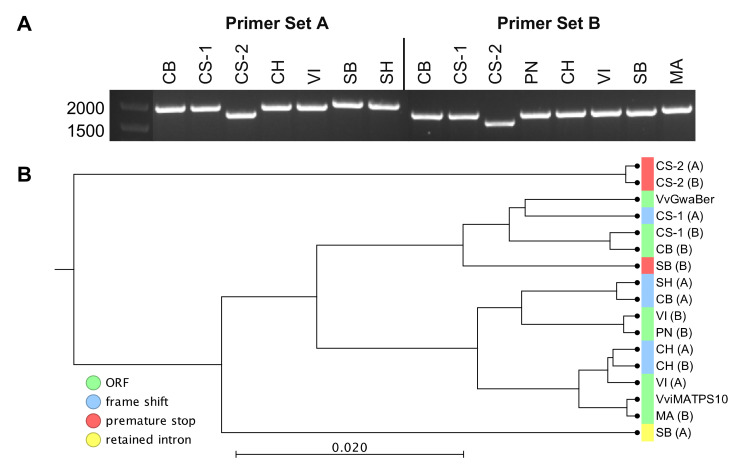
cDNA sequences isolated from different genotypes using the two different primer combinations shown (**A**). Phylogenetic positions relative to the *VvGWaBer* and *VviMATPS10* nucleotide sequences are shown (**B**). The letter code in parentheses refers to the primer set used for isolation with the type of disruption indicated when non-functional.

Isolation of the genes from grapevine flower cDNAs resulted in the cloning for six out of nine genotypes using primer set A and seven out of nine genotypes using primer set B. No amplification occurred from WR. CS resulted in two amplified sequences for both primer sets. The cDNA amplicon sizes were, however, different for the genotypes (Figure 3A). Sequencing confirmed that two different forms were isolated and that the 27bp differences were sufficient for the isolation of genotypic variants, as evident from the pairwise sequence alignments in Supplementary File S2. Of the 15 isolated sequences, only six had fl-ORFs, while the remaining nine genotypic variants were rendered non-functional due to premature stop codons, frame shifts, and/or intron retention (Figure 3B). The MSA for Figure 3B is available in Supplementary File S3.

### 2.3. Transient Expression of fl-ORFs of VviTPS10 in Tobacco

**Figure 4:**
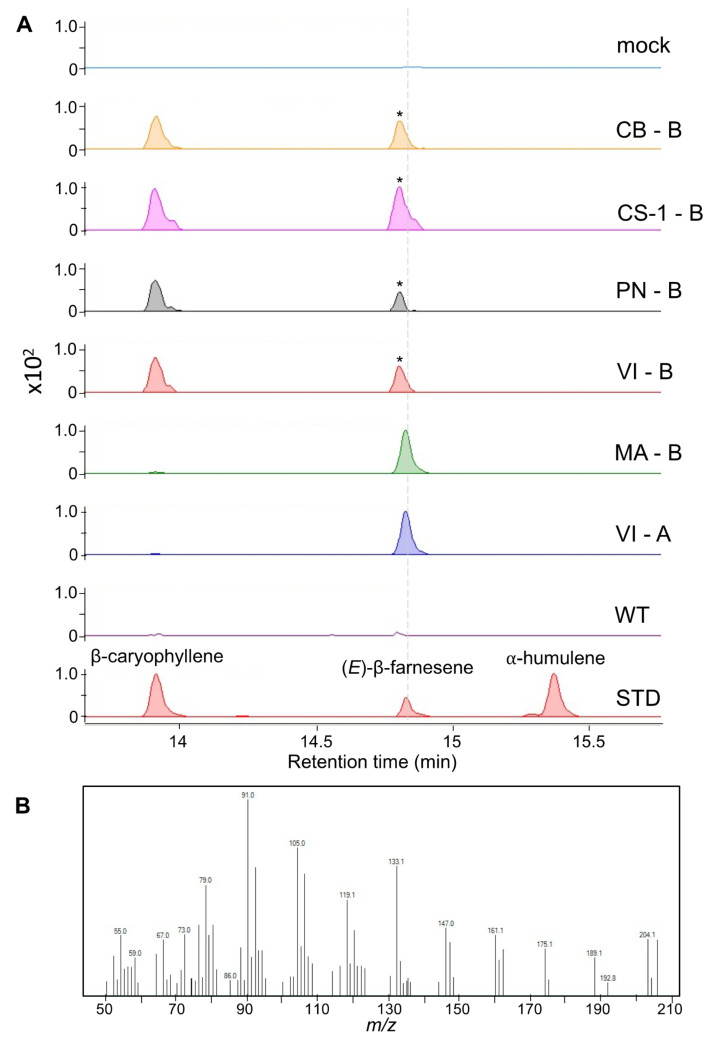
(**A**) Cumulative EIC using the masses 161, 189, and 204 for *N. benthamiana* transiently expressing genes of interest. Cultivar-specific genes are shown with peaks identified using authentic standards (STD). Wild-type (WT) and mock infiltrations served as controls. The *m/z* spectra for the peaks indicated by the asterisk are shown (**B**), with a dashed grey line showing that its retention time is different to that of (E)-β-farnesene. The following cultivar abbreviations were used: Chenin Blanc (CB), Cabernet Sauvignon (CS), Muscat of Alexandria (MA), Pinot noir (PN), and Viognier (VI).

The transient expression in *N. benthamiana* showed that all fl-ORF VviTPS10s produced volatile sesquiterpenes. Isolate VI-A was the only functional gene, other than MA-B (*VviTPS10*), which produced (*E*)-β-farnesene as a single product. The VI genotype was the only cultivar that resulted in a functional gene using both primers, with VI-B producing β-caryophyllene and an unidentified sesquiterpene (Figure 4). The unidentified sesquiterpene had a retention time very near to that of (*E*)-β-farnesene, although its *m*/*z* pattern confirmed that it was not (*E*)-β-farnesene. No definitive match in either the Wiley 275 or NIST libraries could be found due the signal-to-noise ratio of this peak being below the limit of detection (LOD). All other genotypes produced β-caryophyllene and the unidentified sesquiterpene.

### 2.4. Sequence–Function Relationships of Isolated Paralogues

**Figure 5:**
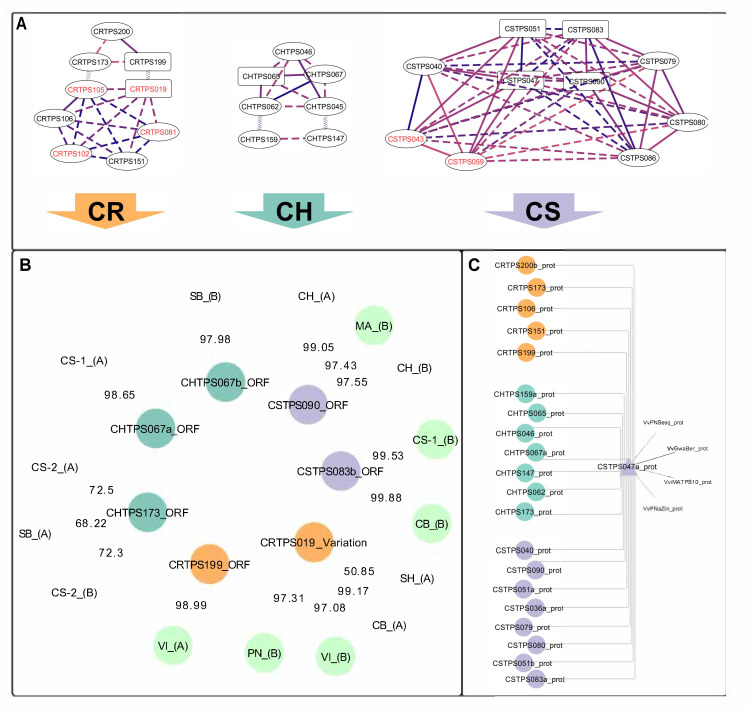
*VviTPS* network connectivity [27] for the paralogues targeted in this study. Genotype-specific duplication networks are shown (**A**). The best-scoring complete gene match of the isolated genes is shown (**B**), with the edge values representing the *I′* score. (**C**) The protein cluster associated with the isolates. The following cultivar abbreviations were used: Chenin Blanc (CB), Chardonnay (CH), Carménère (CR), Cabernet Sauvignon (CS), Muscat of Alexandria (MA), Pinot noir (PN), and Viognier (VI).

Querying the phased diploid *VviTPS* models [27] for the *VviTPS10* paralogues revealed that they are part of a cluster consisting of 21 putative proteins that connect to four functionally characterised proteins (Figure 5C). The amino acid sequence similarity of the active sites to that of the characterised enzymes [7,19,20] showed that the proteins in this cluster are predicted to use NPP as the initial substrate, with all, except VviMATPS10, predicted to have an initial 1,6-cyclisation, as predicted by reference [27]. A subsequent analysis of the associated sequences (Figure 5A) showed that these proteins are encoded by a set of genotype-specific duplicated genes. These duplications form highly connected networks, illustrating the extent of paralogy, with the most extensive network connectivity (i.e., greatest number of putative duplications) seen in CS (Figure 5A). Complete gene models associated with the gene duplicates *CSTPS083* and *CSTPS090* were the closest paralogues of the functional isolates MA-B, CS-1-B, and CB-B (Figure 4B).

**Figure 6:**
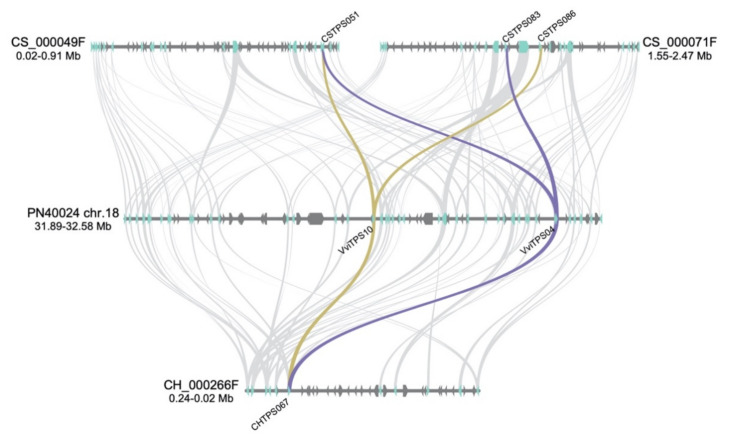
Gene synteny of the *VviTPS* landscape for the Cabernet Sauvignon (CS) and Chardonnay (CH) contigs relative to the PN40024 chr. 18 *VviTPS* cluster. The arrows on the contigs/chr.18 show the direction of the genes, with the *VviTPS* genes shown in light blue. The genes on the CS and CH contigs that are orthologous to PN40024 *VviTPS04* and *VviTPS10* are connected by purple and gold syntenic lines, respectively.

Genomic regions where the duplication clusters (Figure 5A) are located were analysed for gene synteny with the differences in the *VviTPS* landscape of the genotypes, as shown in Figure 6. *VviTPS04* had synteny to two genes located on distinct CS contigs, namely *CSTPS051* and *CSTPS083*. *VviTPS10* shared synteny with *CSTPS051*, while it also showed synteny to *CSTPS083*. *CSTPS083* and *CSTPS086* are 0.12-Mb apart with three genes in between, two of which are *VviTPS* genes, in contrast to *VviTPS04* and *-10*, which are 0.27-Mb apart, with seventeen genes in between them, seven being *VviTPS* genes. A single CH gene, *CHTPS067*, had synteny to both *VviTPS04* and *-10*. The gene synteny analysis was therefore in agreement with the protein sequence homology represented in Figure 5A.

*CRTPS019* and *CRTPS199* were identified as potential gene duplications in CR, with the former not predicted to encode for a functional gene. These two gene models were, furthermore, located on a primary contig and haplotig, respectively, that, unlike the CH and CS paralogues, map to two different chromosomes (chr. 13 and 18). For this reason, we did not include CR for the synteny analysis.

The functional genes PN-B and VI-B had the highest *I*’ scores with the *CRTPS019* gene model (97.31 and 97.08, respectively), while VI-A was linked to *CRTPS199* with a score of 98.99 (Figure 5B). The *I*’ score [38] quantifies the extent of paralogy with phylogenetically similar genes (Figure 4B), linking to distinct proteins based on this score (Figure 5B). This allowed for inference of the protein and gene structures most similar to the isolates.

**Figure 7:**
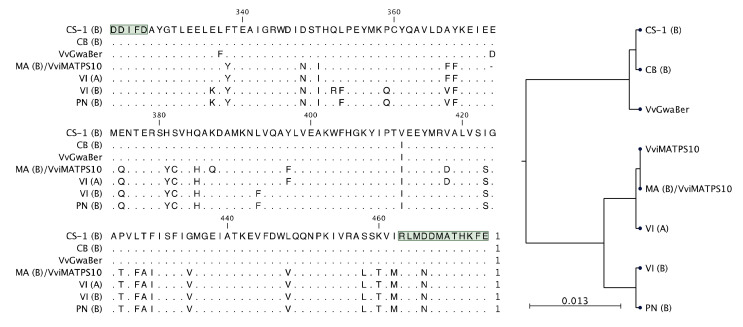
Active site alignment of the six functional isolates to VviMATPS10 and VvGwaBer are shown in (A). The position of the DDxxD and NSE/DTE active site motifs are highlighted in green. The active site similarity is shown in the phylo-genetic tree (B). The following cultivar abbreviations were used: Chenin Blanc (CB), Cabernet Sauvignon (CS), Muscat of Alexandria (MA), Pinot noir (PN), and Viognier (VI).

Although five of the isolates were connected to CH gene models, none of these isolates were predicted functional (Figure 3B). A closer inspection of the active site amino acids of derived protein sequences revealed that VI-A and MA-B, (*E*)-β-farnesene synthases, had near-identical active sites, with the only difference to MA-B being an amino acid deletion and a single nonsynonymous mutation, shown in Figure 7. Furthermore, pairwise comparisons of the full-length proteins for VI-A, VI-B, and MA-A revealed that these proteins were more similar between cultivars than within a cultivar (Supplementary File S4). The active sites of CB-B and CS-1-B were very similar to that of VvGwaBer, with only two amino acid differences (Figure 7). The heterologous in planta expression (Figure 4A), however, did not result in the synthesis of any compounds similar to those observed in vitro for VvGwaBer [7].

## 3. Discussion

### 3.1. Genotype-Specific Structural Variation for Isolated VviTPS Paralogues

Genotypic variations in VviTPS-encoding genes were suggested to have a significant impact on the genetic potential of a given cultivar [20]. By targeting two paralogues in nine genotypes, the extent of the structural differences and sequence variations became evident (Figure 1 and Figure 2). Previous efforts focussed only on the enzyme function, with only two studies reporting on the isolation of new and/or cultivar-specific *VviTPS* genes [19,39]. For example, two point mutations in *VviTPS24* were linked to an altered function in Shiraz [19]. The use of a single reference genome in an “applies-to-all” manner is therefore limiting when trying to explore both sequence and structural variations for VviTPS-encoding genes. It was previously suggested that the genotype-specific landscape of VviTPS-encoding genes is greatly underrepresented in the reference genome when compared to next-generation phased diploid genomes [27]. Comparative analyses of the reference [1,21] and diploid genomes [24,25,26] indicated that genotype-specific SV and/or allelic differences likely resulted in a cluster of *VviTPS* genes on chr. 18 [27]. The results presented here provided functional validation in multiple genotypes for two paralogues in this cluster, namely *VviTPS04* and -*10*.

Once assembled, the diploid genomes should provide new scaffolds for the mapping and mining of SV and SNVs [33,37,40,41,42,43,44] to comprehensively study the allelic differences that impacted the VviTPS functionality. The WGRS data provided valuable insight into the events that shaped the genotypic variations [37,40]. The generation of consensus sequences using gDNA models predicted from phased diploid genomes is advantageous for studying expanded gene families, especially those involved in specialised metabolism. The surveying of WGRS data for 11 different genotypes resulted in 147 consensus sequences with >89% identity to the *VviTPS* gDNA models predicted from the phased diploid [24,25,26] and reference genomes [1,21]. Although the *VviTPS04* and *VviTPS10*-like sequences formed a distinct clade (Figure 1), extensive conservation at the terminal ends (Table 1 and Figure 1) presented a challenge when designing primers to isolate these genes. The in silico analysis of the primer-binding regions provided important insight into the terminal sequence conservation of the targeted paralogues. The consensus sequences, furthermore, allowed us to identify a phylogenetic clade that is specific to *VviTPS04* and *-10*. This reduced the number of gDNA models that could likely encode for these two genes. This greatly increased the available resolution in terms of sequence information, where we previously relied largely on the PN40024 reference genome [1,21]. This approach can be applied to identify and cross-reference *VviTPS* genes of interest in cultivars for which no genome sequence exists. The subtle impact of SNPs, which can render a gene non-functional or alter its catalytic mechanisms, can therefore be explored with a finer focus.

The isolated paralogues had extensive homology at the terminal ends; however, when aligned to the aforementioned gDNA sequences, the isolates showed homology to potentially different genomic regions (Figure 5B). The current lack of assembly for the phased diploid genomes does not allow for definitive chromosome positioning. The advantage of PacBio sequencing does, however, allow for the analysis of large contigs. We previously mapped *VviTPS*-containing contigs to chromosomes using PN40024 as a reference, which allowed for the extrapolation of chromosome localisation [27]. The genotype-specific expansion of *VviTPS04* and *VviTPS10*-like paralogues (Figure 5A and Figure 6) suggests that they are in a chr. 18 region that consists of multiple recent duplications that went through genotype-specific neo-functionalisation, likely exacerbated by domestication and continuous vegetative propagation [41,45].

The CS regions where these paralogues are found (Figure 6) revealed that neither of the primary contigs had a gene order similar to that of PN40024. Assuming that phased sequencing of the two CS contigs in Figure 6 accurately represents two separate genomic regions, one can conclude that these two *VviTPS* regions are part of a much larger (~950 kb) segmental duplication. Alternatively, these two contigs could represent heterozygous allelic regions on chr.18. The misidentification of allelic regions is a limitation of FALCON-UNZIP (the haplotype aware sequence assembler of PacBio sequences), where heterozygous alleles are assembled into primary contigs instead of haplotigs. New algorithms (for example, FALCON-PHASE) have, however, been developed to address this [46]. Whether these are heterozygous alleles or segmental duplications does not change the fact that there are distinctly different genomic regions associated with the *VviTPS* clusters from the different genotypes. The lack of assembled genomes is, however, temporary and will likely be addressed in future.

It was evident that the presence of a transcript does not equate to a functional enzyme, with only 6/15 genes shown to be functional (Figure 3 and Figure 4). Extensive SV between genotypes rendered most isolates non-functional; however, distinct active site forms were identified (Figure 7). The isolation of a second genotype with an active site similar to that of VviMATPS10 [20] provided evidence of a unique active site for (*E*)-β-farnesene biosynthesis in the grapevine. It is not yet clear which amino acid differences are key to this function. The paralogue sequences therefore provided a biologically relevant sequence reference for possible future studies focussed on modelling the protein structure to identify key functional residues for site-directed mutagenesis.

The sequence differences in the terminal end could be used to isolate two distinct loci; however, the VI and MA variants involved in (*E*)-β-farnesene biosynthesis were isolated with different primer pairs. From this, it was concluded that the 27-bp difference used to distinguish paralogues had no significant impact on the enzyme function. The N-terminal region of a TPS is generally accepted to play a structural role (i.e., correct folding of the protein), while the C-terminal, which contains the metal-binding motifs, is considered to be the catalytic region. It is evident from the active site alignment (Figure 7) that two distinct catalytic sites are associated with the functional isolates. VvGwaBer results in α-bergamotene as the major product (56%), with minor amounts of nerolidol, (*E*)-β-farnesene (8%), (*Z*)-α-farnesene (5%), and an unknown terpene (14%) [7]. The catalytic site of VviMATPS10, producing 100% (*E*)-β-farnesene, is likely more constrictive and/or provides less protection from a nucleophilic attack, resulting in earlier quenching. The evolutionary origin of this function is yet to be determined and will require further analysis of the chromosome structure.

### 3.2. The Impact of Domestication on VviTPS Expansion

It is theorised that the diversity in natural terpenes provides a fitness advantage with different models proposed for the origin of this diversity, as reviewed in Pichersky and Raguso (2018) [47]. These models, however, do not account for the influence of domestication within a species; instead, they focus on species-wide evolution. The majority of commercial grapevine cultivars have a shared ancestry and are maintained primarily through vegetative propagation to preserve the desired phenotypic traits [48].

The grapevine genomic structure has been shown to be heavily impacted by somatic variations [45,49]. Of particular interest is the occurrence of chromosome shattering (chromothripsis), where large parts of chromosomes are deleted, resulting in the hemizygous loss of large genomic regions, or incorrect joining of such regions, resulting in clustered rearrangements. This phenomenon was linked to berry colour development, where the hemizygous loss of a genomic region, coupled with specific mutations in regulatory genes of anthocyanin biosynthetic genes, resulted in white grapes [49]. Various studies have quantified the extent of SV and/or SNVs between grapevine genotypes [30,44,50], with a comparison between wine and table grapes showing up to 8% of the genome being affected [51]. The *VviTPS* family has likely been subjected to multiple genome level events, which include segmental duplications [30] and transposon-mediated duplications, evident by the presence of transposon-like remnants around *VviTPS* genes [7].

The *VviTPS* genomic landscape of chr. 18 (Figure 6) suggests that large-scale genome rearrangements [49], compounded by introgression (between species) and admixture (within species) [34,41,52], resulted in heterozygous clustered duplications of *VviTPS* genes. The paralogue/duplication networks in Figure 5A illustrate how these large-scale genomic events resulted in numerous genes of a similar function being localised in a clustered manner. When one considers these clusters in the context of adaptive introgression, where large genomic regions are introduced from a different species, it is highly likely that geographic expansion resulted in the incorporation of alleles adapted to specific environments [34,53]. These alleles have been subjected to extensive mutational changes due to vegetative propagation, allowing for genotype-specific functionality (Figure 3 and Figure 4). It is therefore highly likely that a combination of duplications, chromothripsis, extensive introgression, and SNV events resulted in genotype-specific *VviTPS* clusters, with the primary assemblies of the CH and CS genomes (Figure 6) supporting this hypothesis. In this study, two paralogues were explored and, although limited, provided valuable insight into how the genomic landscape can shape a cultivar’s potential to produce terpenes. Zhou et al. (2017) suggested that clonal propagation results in the accumulation of deleterious mutations on the recessive allele, which could explain the SNV observed that rendered nine of the 15 *VviTPS* genes non-functional. Clonal differences within a single cultivar therefore adds even further complexity to mutational events that impact gene functions [24,54].

### 3.3. VviTPS04 and -10 Functions: An Ecophysiological Perspective

Continuous vegetative propagation ensures that desired traits are maintained but simultaneously provides a relatively unchanging host for pathogens and pests. In order to maintain fruit quality and production levels, European growers apply between 12 and 30 fungicide and 1–10 pesticide treatments per season, depending on the region where the vines are cultivated [55]. *VviTPS04* and -*10* paralogues are of particular ecophysiological interest due to their role in the biosynthesis of (*E*)-β-farnesene and (*E*)-β-caryophyllene, both known kairomones for the grapevine berry moth *Lobesia botrana* [56,57,58]. This moth causes widespread production losses for grapevines planted in the Mediterranean region, with recent reports of the polyphagous relative *L. vanilana* causing damage to grapes in a geographically localised region in the Western Cape of South Africa [59]. Due to the commercial losses caused by the grape berry moth, alternative methods to disrupt the kairomone cues through genetic engineering of grapevines was explored by the overexpression of *Aaβ-FS*, an (*E*)-β-farnesene synthase orthologue from *Artemisia annua* [58,60]. The results from this study were promising, as it decreased moth attraction to grapevines. The isolation and functional characterisation of a grapevine (*E*)-β-farnesene synthase [20] therefore presents a new target for silencing and/or overexpression studies aimed at disrupting *L. botrana* kairomones. (*E*)-β-caryophyllene is, however, a second major grapevine sesquiterpene that, along with (*E*)-β-farnesene and the homoterpene (*E*)-4,8-dimethyl-1,3,7-nonatriene (DMNT), form the core volatiles that attract the grapevine berry moths [56,57,58]. Eight genes, including the ones from this study, have so far been linked to (*E*)-β-caryophyllene biosynthesis in grapevines [7,20], which will make it difficult to create a null background for this sesquiterpene.

## 4. Conclusions

Genome-wide and gene-specific variations resulted in the expansion of the *VviTPS* family, resulting in tremendous genetic diversity. The paralogues characterised in this study provided valuable insight into how SV and SNP impact gene functions within and between genotypes. WGRS data coupled with the increasing number of diploid *Vitis* spp. genome sequences will undoubtedly provide valuable new genetic targets to study *VviTPS* gene variations. Our integration of WGRS with sequenced diploid genomes allowed for an in-depth analysis of the genotypic differences for loci believed to be duplications. Furthermore, we showed that there are extensive gene synteny differences for the CS and CH genotypes when compared to the *VviTPS* gene cluster on chr. 18 of the PN40024 reference genome. This provides further evidence for the need to move beyond the use of a single reference genome when studying expanded gene families.

The complex biomechanical aspects of isolated *VviTPS* genes were shown through functional characterisation of the paralogues. Two distinct active sites were identified with transient heterologous expressions showing different biochemical properties for the genotypic isolates. By utilising genomic and WGRS resources to consider both the extent of paralogy and heterozygosity, it should now be possible to more accurately target *VviTPS* genes to establish structure–function relationships for specific genotypes. Extending our methodology to other expanded gene families—for example, cytochrome P450s—will allow for the identification of genotype-specific variations and/or novel genes.

## 5. Materials and Methods

### 5.1. Identification of Putative Paralogues Using Diploid Grapevine Genomes

*VviTPS04* and -*10* gene models are highly homologous on the PN40024 genome, positioned 262 kb apart on chr18 [7,21]. *VviTPS10* was previously shown to have multiple putative paralogues on the Cabernet Sauvignon (CS) genome [20]. The analysis of the paralogues was extended to the Chardonnay (CH) and Carménère (CR) genomes, and the CS genome was re-evaluated using the respective curated gene models reported by reference [27]. The curated *VviTPS04*- and -*10* genomic sequences [7] were retrieved from FLAGdb++ [61] and queried with BLAST [62] to identify their homologous gDNA sequences in the phased diploid genomes.

### 5.2. Identification of Putative VviTPS04 and -10 Structural Differences Using Whole-Genome Resequencing Data

Short-read sequencing data of eleven *Vitis* accessions were retrieved from the Sequence Read Archive (SRA) under BioProject ID PRJNA388292 [37]. SRA data was converted to the FASTQ format using the SRA Toolkit (https://github.com/ncbi/sra-tools (Accessed on 25 June 2020)). Magic-BLAST [63] was used to map short reads to genomic reference sequences. The resulting Sequence Alignment Map (SAM) files were analysed using Geneious Prime v2020.1.2. (Biomatters Ltd., Auckland, New Zealand) The 330 SAM mappings generated were manually assessed to ensure sufficient coverage relative to the reference sequence, with no internal gaps in coverage, followed by the extraction of consensus sequences. The CLUSTAL-Omega algorithm [64,65] was used for multiple sequence alignments (MSA) using Geneious Prime v2020.1.2. (Biomatters Ltd., Auckland, New Zealand. The subsequent phylogenetic tree was constructed using IQ-TREE 2 [66] with extended model selection followed by tree inference (-m MFP), 1000 ultrafast bootstrap replicates (-B 1000), 1000 replicates for single-branch testing (--alrt 1000), and ‘DNA’ specified as sequence type (--seqtype DNA). The phylogenetic tree was visualised and further modified using iTOL (https://itol.embl.de/, accessed on 14 June 2021) [67].

### 5.3. Isolation and Cloning of VviTPS04 and -10 Paralogues

Primers were designed to target putative paralogues: forward primer A (*VviTPS10* paralogues): 5′-ATGGCCTTAATTCTCGCTACCAGCAACGGG-3′, forward primer B (*VviTPS04* paralogues): 5′-ATGTTGAGTGCTCGAGTGAGTTTACACATGGCC-3′), and a single conserved reverse primer: 5′-TCATATTGGCACAGGGTCTATGAGCAGCATTGAAATATT-3′.

RNA extractions from 100-mg flower tissue of nine *V. vinifera* genotypes were performed using the Spectrum Plant Total RNA Kit (Sigma-Aldrich, St. Louis, MO, USA) with an on-column DNase I (Thermo Fisher Scientific, Waltham, MA, USA) treatment. RNA was checked for genomic DNA (gDNA) contamination through PCR, followed by reverse strand synthesis (cDNA) using the SuperScript VILO Master Mix (Thermo Fisher Scientific, USA). Genomic DNA (gDNA) was isolated from the same genotypes according to the method described by reference [68]. RNA extractions from 100-mg flower tissue of nine *V. vinifera* genotypes were performed using the Spectrum Plant Total RNA Kit (Sigma-Aldrich, St. Louis, MO, USA) with an on-column DNase I (Thermo Fisher Scientific, Waltham, MA, USA) treatment. RNA was checked for genomic DNA (gDNA) contamination through PCR, followed by reverse strand synthesis (cDNA) using the SuperScript VILO Master Mix (Thermo Fisher Scientific, Waltham, MA, USA). Genomic DNA (gDNA) was isolated from the same genotypes according to the method described by reference [68]. Flower genotypes were collected at stage 17/18 according to the modified Eichhorn and Lorenz (EL) growth classification system [69] for the following cultivars: Chardonnay (CH), Chenin Blanc (CB), Muscat of Alexandria (MA), Pinot noir (PN), Pinotage (PI), Sauvignon Blanc (SB), Shiraz (SH), Viognier (VG), and Weisser Riesling (WR). Samples were a composite of six to eight flowers clusters that were frozen in liquid nitrogen, with the rachis structures separated before homogenisation. All genotypes were planted in close geographical proximity at a mother block in the Stellenbosch area (33°57′33.50″ S, 18°51′38.09″ E), South Africa.

PCR reactions using cDNA or gDNA as the template was performed using TaKaRa ExTaq proofreading polymerase, as per the product specifications (Separations, Roodepoort, South Africa). PCR reaction conditions were as follows: 2 min initial denaturation, followed by 35 cycles of 30 sec denaturation, 30 s annealing, 2 min of 30-s extension, and a final extension of 7 min. cDNA amplicons were purified after electrophoretic separation using the Zymoclean Gel DNA Recovery Kit (Inqaba Biotech, Pretoria, South Africa), TA-cloned, screened for positives through colony PCR, and the plasmids isolated from overnight cultures. Plasmid DNA was diluted to 1/50 and used as a template for two-step Gateway PCR with the primers modified accordingly to build in *attB* sites, as described in the Gateway Technology Manual (Thermo Fisher Scientific, Waltham, MA, USA). Gateway cloning was performed according to the aforementioned manual using the appropriate clonases to generate entry clones with pDONR-Zeocin (Thermo Fisher Scientific, Waltham, MA, USA) and expression clones with pEAQ-HT-DEST1 [70,71]. The respective clones were transformed into chemically competent DH5α *Escherichia coli* using appropriate antibiotics and screened for positives through colony PCR. Entry clones were bidirectionally sequenced according to the standard methods of the Central Analytical Facility, Stellenbosch University, South Africa using M13 sequencing primers and a walking primer (5′-CTCTTCTATTGTTGGTATGTATTTTC-3′).

### 5.4. Sequence Analysis of Isolated Paralogues

Sequence analyses were performed using CLC Main Workbench 7 (CLC Bio-Qiagen, Aarhus, Denmark). Phased diploid annotations of the *VviTPS* family [27] were queried to identify the cluster of proteins most similar to the sequenced isolates. MUSCLE alignments [72,73] and phylogenetic tree construction were performed as described earlier using CLC Main Workbench 7 (CLC Bio-Qiagen, Aarhus, Denmark). Gene sequence data and Cytoscape network data were retrieved, as per the instructions in reference [27]. The *I′* score [38] was calculated from BLASTn [62] scores of isolates queried against all diploid complete *VviTPS* genes (mRNA sequences) predicted by reference [27].

### 5.5. Gene Synteny Analysis

The MCScan pipeline [74] in the JCVI utility library [75] was used to perform a gene synteny analysis. Coding sequences for genes on contigs VvCabSauv08_v1_Primary000071F, VvCabSauv08_v1_Primary000049F [26], 000266F of Chardonnay [24], and PN40024 chr. 18 [1,21] were retrieved. The corresponding gene annotations were used to parse the GFF files to the BED format required for jcvi:MCScan. The gene synteny (microsynteny) analysis and visualisation were performed according to the instructions in the JCVI utility library using the default settings.

### 5.6. Agrobacterium-Mediated Transient Expression

The plasmids listed were transformed via electroporation into the *Agrobacterium tumefaciens* GV3101::pMP90 strain, as described by reference [76], and plated onto selective media. *Agrobacterium*-mediated transient expression in *Nicotiana benthamiana* was performed, as described by reference [20], with three leaves from a single plant pooled after four days for the subsequent analysis. A mock infiltration using MMA buffer and non-infiltrated wild-type leaves served as controls. Headspace solid-phase microextraction (HS-SPME) and gas chromatography-mass spectrometry (GC-MS) was performed as described by reference [77], with the following modifications: adsorption of analytes was done at 30 °C for 20 min and an initial oven temperature of 100 °C. Sesquiterpenes were identified using the authentic standards (*E*)-β-farnesene and (*E*)-β-caryophyllene from Sigma-Aldrich, USA and/or the Wiley 275 mass spectral library. For extracted ion chromatograms (EIC), the cumulative mass (*m*/*z*) response for 161, 189, and 204 was used.

## Figures and Tables

**Table 1:** Consensus sequences assembled from WGRS data [37] using *VviTPS* gDNA models predicted from phased diploid genomes [27] for the cultivars Chardonnay (CH) [24], Carménère (CR) [25], and Cabernet Sauvignon (CS) [26] and the grapevine reference genome PN40024 [1,7]. Cultivars used in the mapping are indicated with their respective accessions, indicated in parentheses. Consensus sequences with a binding site for either primer A (green) or primer B (red) are shown, with group C (orange) indicating sequences that do not have coverage at the 5′-terminal ends.

gDNA Model	Genome	Cabernet Franc (TA-183)	Carménère (TA-254)	Cabernet Sauvignon (TA-280)	Chardonnay (TA-238)	Merlot (TA-241)	Muscat of Alexandria (TA-170)	Pinot Noir (TA-379)	Riesling (TA-329)	Sangiovese (TA-334)	Sauvignon Blanc (TA-417)	Syrah (TA-242)
*CHTPS045*	CH				A					A		
*CHTPS046*	CH	A	A	A	A	C	A	A	A	A	A	A
*CHTPS062*	CH		B	B	B							
*CHTPS065*	CH				C		A	A	A			A
*CHTPS067*	CH				B			C	C	C		B
*CHTPS147*	CH	A	A	A	A	A		A	A	A	A	A
*CHTPS159*	CH				B							
*CHTPS173*	CH	C	C	C	C	C	C	C	C	C	C	C
*CRTPS019*	CR	A	A	A		A			A	A	A	A
*CRTPS081*	CR	A	A	C		A			C	A	A	A
*CRTPS102*	CR											C
*CRTPS105*	CR											
*CRTPS106*	CR	A	A	A		A		A	C		A	C
*CRTPS151*	CR	A	A	A	A	A	A		A	A	C	A
*CRTPS173*	CR											A
*CRTPS199*	CR						A	A	A			A
*CRTPS200*	CR				B			C	C			B
*CSTPS036*	CS		A	A					A		A	A
*CSTPS040*	CS	A	A	A	A	A	A		A	A	C	A
*CSTPS043*	CS											A
*CSTPS047*	CS		C	B				C	C			
*CSTPS051*	CS		B	B	B							
*CSTPS059*	CS		A	A								C
*CSTPS079*	CS		A	A					C	A		A
*CSTPS080*	CS			A	A		A	A	A	A	A	A
*CSTPS083*	CS				C							
*CSTPS086*	CS				B		B					
*CSTPS090*	CS				C		A		A			
*VviTPS04*	PN40024		B	B	B			B				
*VviTPS10*	PN40025				C		A	C	A		A	C

## Data Availability

The data presented is contained within article and Supplementary Files.

## Supplementary Materials

The following are available online at https://www.mdpi.com/article/10.3390/plants10081520/s1: Supplementary Figure S1: Complete phylogenetic tree of the gene models predicted from WGRS mappings with the primer bindings indicated. Supplementary File S1: Nucleotide sequences for reference genes and those predicted by mapping of the WGRS data. Supplementary File S2: Pairwise alignments of genotypic variants isolated from different *V. vinifera* genotypes. Supplementary File S3: Amino acid alignments of the phylogenetic tree shown in Figure 3B. Supplementary File S4: Amino acid alignments of the functional genotypic variants.
