# Supplementary material for: Seeing the Forest through the (Phylogenetic) Trees: Functional Characterisation of Grapevine Terpene Synthase (VviTPS) Paralogues and Orthologues
Source: Plants (Basel). 2021 Jul 26;10(8):1520. doi: 10.3390/plants10081520 (PMC8401418; doi:10.3390/plants10081520)

Figure S1: Complete phylogenetic tree of the gene models predicted from WGRS mappings with the primer bindings indicated

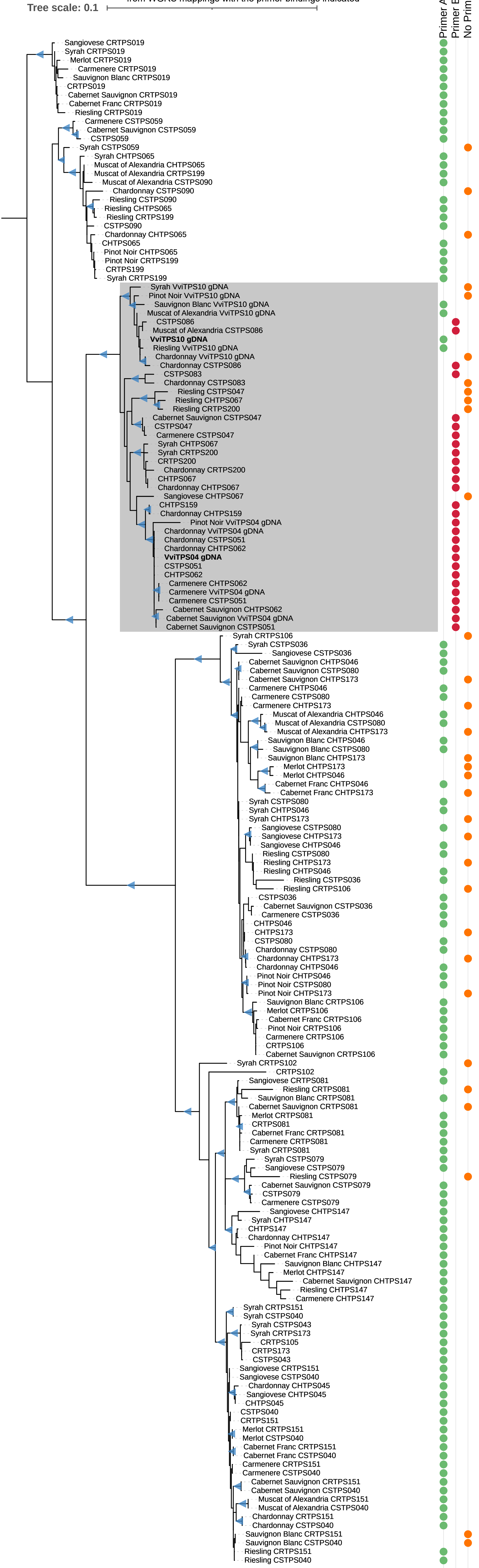

Supplement: Supplementary file 1 [file plants-10-01520-s001.zip › plants-1288915-supplementary/Figure S1.pdf]
